# A Galactooligosaccharide Product Decreases the Rotavirus Infection in Suckling Rats

**DOI:** 10.3390/cells11101669

**Published:** 2022-05-18

**Authors:** Malén Massot-Cladera, María del Mar Rigo-Adrover, Laura Herrero, Àngels Franch, Margarida Castell, Jelena Vulevic, Francisco J. Pérez-Cano, María J. Rodríguez Lagunas

**Affiliations:** 1 Departament de Bioquímica i Fisiologia, Facultat de Farmàcia i Ciències de l’Alimentació, Universitat de Barcelona (UB), Av. Joan XXIII 27-31, 08028 Barcelona, Spain; malen.massot@ub.edu (M.M.-C.); m.rigo@ub.edu (M.d.M.R.-A.); lherrero@ub.edu (L.H.); angelsfranch@ub.edu (À.F.); margaridacastell@ub.edu (M.C.); mjrodriguez@ub.edu (M.J.R.L.); 2 Institut de Recerca en Nutrició i Seguretat Alimentària (INSA), C/Prat de la Riba 171, 08921 Santa Coloma de Gramanet, Spain; 3 Institut de Biomedicina de la Universitat de Barcelona (IBUB), Universitat de Barcelona, 08028 Barcelona, Spain; 4 Centro de Investigación Biomédica en Red de Fisiopatología de la Obesidad y la Nutrición (CIBEROBN), Instituto de Salud Carlos III, 28029 Madrid, Spain; 5 veMico Ltd., Reading RG6 6AR, UK; jelena@vemico.co.uk

**Keywords:** galactooligosaccharides, rotavirus, diarrhea, infection

## Abstract

The leading cause of gastroenteritis among young children worldwide is the Group A rotaviruses (RV), which produce a wide range of symptoms, from a limited diarrhea to severe dehydration and even death. After an RV infection, immunity is not complete and less severe re-infections usually occur. These infections could be ameliorated by nutritional interventions with bioactive compounds, such as prebiotics. The aim of this research was to study the impact of a particular galactooligosaccharide (B-GOS) on the RV symptomatology and immune response during two consecutive infections. Lewis neonatal rats were inoculated with SA11 (first RV infection) on day 6 of life and with EDIM (second RV infection) on day 17 of life. B-GOS group was administered by oral gavage with a daily dose of B-GOS between days three to nine of life. Clinical and immunological variables were assessed during both infective processes. In the first infection, after the prebiotic intervention with B-GOS, a lower incidence, duration, and overall severity of the diarrhea (*p* < 0.05) was observed. In addition, it improved another severity indicator, the fecal weight output, during the diarrhea period (*p* < 0.05). The second RV infection failed in provoking diarrhea in the groups studied. The immune response during first infection with SA11 was not affected by B-GOS administration and had no impact on second infection, but the prebiotic intervention significantly increased IFN-γ and TNF-α intestinal production after the second infection (*p* < 0.05). In summary, B-GOS supplementation is able to reduce the incidence and severity of the RV-associated diarrhea and to influence the immune response against RV infections.

## 1. Introduction

Rotavirus (RV) infection is responsible for causing severe acute gastroenteritis worldwide and the children under 5 years old are at greatest risk for developing diarrhea. RVs are double-stranded RNA (dsRNA) with the ability to infect enterocytes of the small-intestinal villi, causing the disruption of absorptive functions and fluid secretion [1,2,3]. This increase in water secretion into the small intestine lumen induced by infection is mediated by the enteric nervous system (ENS) as a mechanism to promote pathogen clearance, thus evidencing the importance of the neuroimmune crosstalk during infection [4]. Because the immune system is immature during the first years of life [5], bioactive components present in breast milk such as immunoglobulins (Ig) or oligosaccharides are important to prevent diarrhea morbidity and mortality during the lactation period [6].

The natural RV infection induces an immune response that leads to the production of specific Ig and the activation of T cells, therefore preventing the invasion of enterocytes and limiting the RV spread. However, reinfections occur frequently in children, but each time showing a milder disease, demonstrating that after the infection there is a partial immune protection against RV, which is not complete until several infections are passed [6,7].

There are no drugs available to treat RV infection and for this reason, the main objective of the treatment is to replace fluids and electrolytes lost due to diarrhea and vomiting. In addition, the commercially available oral, inactivated, liv- RV vaccines to prevent infection such as Rotarix^®^ (GSK) and RotaTeq^®^ (Merck) have demonstrated variable security and efficacy in clinical trials carried out in different countries around the world [8,9]. Moreover, it is well-known that the intake of certain nutritional supplements can improve the state of health and might prevent some diseases [10]. In this regard, there is evidence that the use of probiotics and prebiotics may be effective in preventing and ameliorating the RV diarrheic process [11,12].

Probiotics have proven to be effective in different gastrointestinal disorders with a neuroimmune origin [13,14], however, the role of prebiotics in the pathogenesis of these diseases needs to be further studied. Prebiotics are defined as substrates that are selectively utilized by host microorganisms conferring a health benefit [15].

A variety of different carbohydrates of vegetable and animal origin and with prebiotic activity has emerged through different manufacturing procedures.Fructooligosacharides (FOS) and galactooligosacharides (GOS) are prebiotics commonly found in infant formulas. They promote beneficial changes in stool consistency and in bacterial composition in infants and increase short chain fatty acids (SCFA) and lactate in caecum samples [11,15]. Overall, different mixtures of GOS have demonstrated a preventive effect in the RV-associated diarrhea at both preclinical and clinical levels [16,17]. In line with this, B-GOS (Bimuno GOS, Clasado Biosciences Ltd., Reading, UK), a product manufactured by reverse β-galactosidase synthesis from enzymes in *Bifidobacterium bifidum* NCIMB 41 171 [18], has demonstrated bifidogenic activity in vitro and in human interventions [18,19,20,21,22]. In addition, administration of B-GOS to elderly volunteers also positively modulated some immune function markers [23] and showed anti-infective mechanisms against *Salmonella enterica Typhimurium* [24].

Although diverse animal models of RV infection exist in rodents, those in mice induce severe diarrhea, which do not allow researchers to observe the nutritional prevention, and those in rats are highly specific to few RV and rat strains [7]. In both cases, the main limitation is that the evaluation of the disease severity is based on stool scoring, which can be subjective unless a clear protocol is not followed, and it is not based on the opinion or scoring of various blinded researchers, as well as it is not accompanied by other variables that sustain the results. In addition, although multiple infections could be induced in a model, the symptoms only appear in early life, thus in the first infection [7].

Considering the potential role of this particular prebiotic on infection and considering that its action in early life remains unknown, the present study aimed to stablish its role in the prevention of the RV infection and immunomodulatory capacity in a RV-double-infection neonatal rat model.

## 2. Materials and Methods

### 2.1. Animals

Nine G14 pregnant Lewis rats (LEW/Han^®^Hsd) from Harlan (Horst, The Netherlands) were individually housed (2184L Eurostandard Type II L, Tecniplast, West Chester, PA, USA) in cages that were enriched with large fibrous particle bedding and tissue papers, and monitored daily until the delivery day. The day of birth was considered as day 1. The number of pups was unified to seven in all litters and similar number of each sex was kept in each nest (4:3 or 3:4 males:females in all litters). Rat pups had free access to the dams’ nipples and a solid diet. The rats were housed in controlled conditions of temperature, humidity, and light:dark cycles. Rats were ubicated in a room that was especially designed for this type of approach (biosecurity level 2 conditions), at the Animal Unit of the Faculty of Pharmacy and Food Science from the University of Barcelona (UB). The dams had access to water and a commercial diet (Teklad Global Diet 2014, Envigo, Indianapolis, IN, USA) corresponding to the American Institute of Nutrition 93G formulation [25] *ad libitum*. The experimental procedure was executed in line with the institutional guidelines for the care and use of laboratory animals and was approved by the Ethical Committee for Animal Experimentation (CEEA) of the UB and the Catalonia Government (CEEA-UB Ref.493/12 and, DAAM: 6905, respectively), in full compliance to national legislation following the EU-Directive 2010/63/EU for the protection of animals used for scientific purposes.

### 2.2. Viruses

Animals were infected with two type A viruses at different time points. The simian agent 11 (SA-11) was used for the first infection, and the epizootic diarrhea of infant mouse virus (EDIM) for the second infection. The first virus was produced by the “Enteric Virus Group-UB” [26] and the second one was obtained in vivo by inoculating suckling BALB/c mice (Janvier, La Plaine Saint Denis Cedex, France), following the same procedures of previous studies [7,27].

### 2.3. Experimental Design and Dietary Supplementation

Litters were organized into three different experimental groups: reference (REF), double rotavirus infection (DRI), and RV-infected animals receiving the prebiotic Bimuno GOS (B-GOS). The B-GOS mixture (Clasado Biosciences, Reading, UK) had a 48% GOS content (wt:wt) and its composition in terms of the degree of polymerization (DP) was DP 2 = 52, DP 3 = 26, DP 4 = 14, and DP 5 = 8. The B-GOS saccharide linkages were as follows: β1→3 = 26, β1→4 = 23, β1→6 = 51. Its average molecular weight was 496.8 kDa.

Each experimental group was formed by three litters of seven pups each (*n* = 21/group). The prebiotic administration was orally performed as previously described [28], using low-capacity syringes (Hamilton Bonaduz, Bonaduz, Switzerland) adapted to 25- or 23-caliber forced alimentation tubes that were 27 mm in length (ASICO, Westmont, IL, USA), and was performed from day 3 of life −2 days before the first infection—until day 14 of life −3 days before the second infection-. The B-GOS supplement was administered p.o. at a dose of 0.8 g/100 g of body weight (BW)/day. Rats from the inoculated control group (DRI group) and the non-inoculated control group (REF group) received the same volume of bottled mineral water.

The RV inoculations (SA-11 and EDIM) were performed in the DRI and B-GOS experimental groups, 1 h after the dams’ separation in order to limit the antipathogenic action of the milk components [29]. SA11 was orally inoculated at day 6 of life at a dose of ~2 × 10^8^ TCID_50_ RV/rat and EDIM was inoculated at day 17 at a dose of ~1.3 × 10^8^ RV/rat. Animals were weaned prior to the second RV administration in order to disrupt the pups’ defenses and to facilitate the infection.

Three animals from each litter were euthanized on day 16 of life (to evaluate the impact of the first infection), and the rest on day 28 (to evaluate the host defenses after both RV inoculations). Fecal samples were obtained daily during the study. Some other samples such as blood, small intestine, gut wash (GW), small intestinal tissue and isolated spleen and mesenteric lymph node (MLN) cells were obtained at the end of the study. Body temperature and fecal pH were measured during the post-inoculation periods (days 7–10 and 18–21). The delayed-type hypersensitivity (DTH) response was determined at the end of the study.

### 2.4. Clinical Indexes and Fecal Specimen Collection

The impact of both RV inoculations was evaluated along the study by the BW assessment and the fecal scoring. In addition, fecal samples were immediately weighed and frozen at −20 °C for further analysis. The severity of diarrhea was obtained by scoring stools from 1 to 4 (diarrhea index [DI]) based on their color, texture, and amount, as in previous studies [26]. Values of DI ≥ 2 indicate diarrhea whereas DI < 2 indicate normal feces. Incidence of diarrhea was calculated as the percentage of diarrheic animals (%DA)—which is referred to the number of animals in each group—and, by the percentage of diarrheic feces (%DF)—which takes into consideration the number of total samples collected per day in each group. The area under the curve of severity (sAUC from the day of infection to resolution day) or % DA and % DF (daAUC and dfAUC) were calculated as a global value of the process.

### 2.5. Rectal Temperature and Fecal PH Determination

A TEMP JKT thermometer (Oakton, Vernon Hills, IL, USA) and an RET-3-ISO rectal probe for neonatal rats (Physitemp, Clifton, NJ, USA) were used to measure the rats’ body temperature, using peanut oil (Acofarma, Terrassa, Spain) for lubrication, as in previous studies [7]. The initial temperature was evaluated the day before the RV inoculation (the final temperature was determined 1 day after the RV inoculation and the results are expressed as the increase between them). Fecal specimens from rats during the studied period were diluted in distilled water (up to 200 mg/mL), agitated and then the pH was measured using a 5207 pH electrode for surfaces and a micropH 2001 pH meter (Crison Instruments, Barcelona, Spain).

### 2.6. Viral Shedding

Diluted fecal samples (10 mg/mL in PBS) from the following days after both infections were homogenized using a FastPrep (MP Biomedicals, Santa Ana, CA, USA). Supernatants from centrifuged homogenates (19,000× *g*, 3 min) were frozen at −20 °C until use. Both type of RV particles, from SA11 and EDIM, in fecal samples were quantified by ELISA, as previously described [29]. Serial dilutions (ranging from 4 × 10^5^ to 2.5 × 10^4^/mL) of inactivated SA11 virus particles were used as standard in each plate.

### 2.7. DTH Response

DTH response evaluation was performed as previously described [7,27]. Briefly, the thickness of both the right and the left ear of each animal was measured on day 26 (two days before sacrifice) as being the basal conditions by using a 7309 pocket thickness gauge (Mituyoto, Hampshire, UK). For RV priming, animals were anesthetized with isoflurane (Abbott Laboratories, Berkshire, UK) and a volume of 20 μL of UV-inactivated virus (~0.5 × 10^6^ RV particles/mL) was injected into the right ear, whereas the same volume of saline solution was injected into the left ear. The evaluation of the ear thickness was performed again after 24 h and 48 h. Results are expressed as the thickness increase in the right minus the increase in left ears’ thickness.

### 2.8. Sample Collection

Rats from each group at days 16 or 28 were anesthetized with an intramuscular ketamine/xylazine injection (Imalgene 100 mg/mL, Merial Laboratorios, Barcelona, Spain/Rompun^®^ 20 mg/mL, Bayer Hispania, Sant Joan Despí, Spain). Euthanasia was then performed by opening the peritoneal cavity and disrupting the diaphragm. Blood was collected by cardiac puncture and, after centrifugation, sera was stored at −20 °C. The GW were obtained as in previous studies [7] and stored at −80 °C until analysis. In addition, 1 cm of the middle part of the small intestine was placed in RNAlater (Ambion, Thermo Fisher Scientific, Barcelona, Spain) at −20 °C for the gene expression analysis. The spleen and MLN were removed under sterile conditions and their cells were isolated as previously described [30]. An automated cell counter (Countess™, Invitrogen, Madrid, Spain) was used to assess the cell number and viability. Isolated cells were cultured in a 24-well plate for 72 h under SA11/EDIM mix stimulatory conditions (10^5^ viral particles/mL). After centrifugation the supernatants were collected and kept at −80 °C until cytokine determination.

### 2.9. ELISA and ELISPOT for Anti-RV Antibody Levels and Production

Anti-RV antibody (Ab) levels in serum, GW, and supernatants from the spleen and MLN cell culture were quantified by ELISA, as previously described [27]. Briefly, 96-well plates (Nunc MaxiSorp, Wiesbaden, Germany) coated with UV-inactivated 10^5^/mL SA11 particles and blocked with phosphate-buffered saline (PBS) and 1% bovine serum albumin (BSA, 1 h, room temperature (RT)) were incubated with dilutions of sera or GW samples during 3 h at RT. After washing, polyclonal anti-rat Ig conjugated to peroxidase (Dako, Barcelona, Spain) and the substrate was added. The standard used was a pooled serum from the dams of inoculated litters. The absorbance was measured using a microplate photometer (LabSystem Multiskan, LabX) and analyzed with the ASCENT version 2.6 software (Thermo Fisher Scientific).

An ELISPOT technique was used to quantify anti-RV Ig-secreting cells (SC) from spleen and MLN, as in previous approaches [27], using 96-well nitrocellulose plates (Merck Millipore) coated with 10^5^ particles/mL of viral SA11 or EDIM particles. Each spot obtained corresponded to one anti-RV Ig-SC. Spots were automatically counted by the ELISPOT reader system (AID, Strasberg, Germany) and results are expressed as Ig-SC/10^6^ cells.

### 2.10. Cytokine Analysis by a Bead Immunoassay

IFNγ, IL-4, IL-10, and TNFα concentrations in GW samples and in supernatants from stimulated spleen cells were quantified by a Cytometric Bead Assay Rat Soluble Protein Flex Set (BD Biosciences, Madrid, Spain) as in previous studies [31]. A FacsAria SORP sorter (BD, San José, CA, USA) from the Flow Cytometry Unit (FCU) of the Scientific and Technological Centers of the UB (CCiT-UB) was used. Data analysis was performed using the FlowJo 10.0.7 software (Tree Star, Inc., Ashland, OR, USA).

### 2.11. Real-Time PCR for Small Intestine Gene Expression

At day 28, the small intestine gene expression of receptors from the toll-like receptors (TLR) family (TLR2 and TLR4), Th1 and Th2 responses (IFN-γ and IL-4, respectively), barrier molecules (Occludin, Claudin-2), mucus production (mucin), and regulatory response (IL-10) was studied. For that, homogenates of the tissue samples were obtained by lysing matrix tubes in a FastPrep-24 instrument (MP Biomedicals, Illkirch, France). RNA was isolated with the RNeasy^®^ Mini Kit (Qiagen, Madrid, Spain) following the manufacturer’s instructions as previously described [31]. The amount, purity, and the RNA integrity number were evaluated at the Genomic Service of the CCiT-UB (GS-CCiT-UB). RNA was reverse transcribed, as previously described [31]. PCR Taqman primers and probes specific for rat target genes were obtained from AB. Normalization with HPRT as a housekeeping gene was analyzed using the 2^−^^ΔΔCt^ method [31]. Results are expressed as the mean ± SEM of the percentage of each gene in the DRI and B-GOS groups with respect to the REF group, which represents 100% of gene expression.

### 2.12. Statistical Analysis

The Appraising Project Office’s program from the Universidad Miguel Hernández de Elche (Alicante) was used to calculate the number of animals used in each group, considering the number of individual pups as the statistical sampling unit. Statistically significant differences between groups were considered assuming that there was no dropout rate and type I errors of 0·05 (two-sided). The number of pups used came from at least three litters due to the importance of the litter effect, which has been previously studied [27,32], thus, three litters of 7 animals per group were enough for the sample size estimation that was performed. The final number of animals was not affected by the dropouts or outliers, which did not occur in the current study.

For the statistical analysis, the Statistical Package for Social Sciences (SPSS v.22.0, IBM, Chicago, IL, USA) was used. To assess normal distribution and variance equality, the Shapiro–Wilk test followed by the Levene’s test were used. A conventional one-way ANOVA test was performed, considering the experimental group as the experimental variable. When virus inoculation or dietary interventions had a significant effect on the dependent variable, Scheffé’s post hoc test was applied. When non-normal distribution or different variance was found, the Kruskal–Wallis and Mann–Whitney U tests were used. Finally, to compare frequencies the Chi-squared test was used. Differences were considered significant at *p* values of <0.05. All the results are expressed as mean ± SEM.

## 3. Results

### 3.1. Body Weight

**Figure 1:**
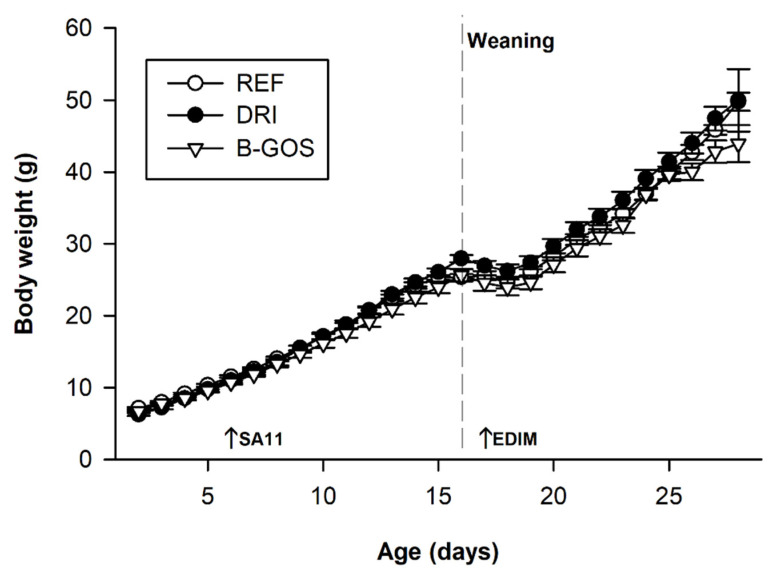
Body weight (g) for reference (REF), double-rotavirus-infected (DRI), and prebiotic-supplemented (B-GOS) animals. SA-11 and EDIM inoculation days (6 and 18, respectively) are indicated by arrows and the weaning day by a dashed vertical line on day 17. Results are expressed as the mean ± SEM of *n* = 12–21 animals/group. Statistical significance is detailed in the text.

The BW was recorded from day two until the end of the study (Figure 1). The initial BW was similar among all groups (about 6–7 g). Neither the SA11 nor the EDIM infection affected the overall BW of the animals, but a weight loss was observed on day 17 in the REF, DRI, and B-GOS groups (*p* < 0.01) due to the early weaning induced in the model. With regard to the effect of the prebiotic supplementation, the BW in B-GOS group was slightly lower than that in the DRI group (*p* < 0.05) during the period of 13–19 days, but at the end of the study (day 28), the BW was similar in all groups.

### 3.2. Incidence and Severity of the Diarrhea

**Figure 2:**
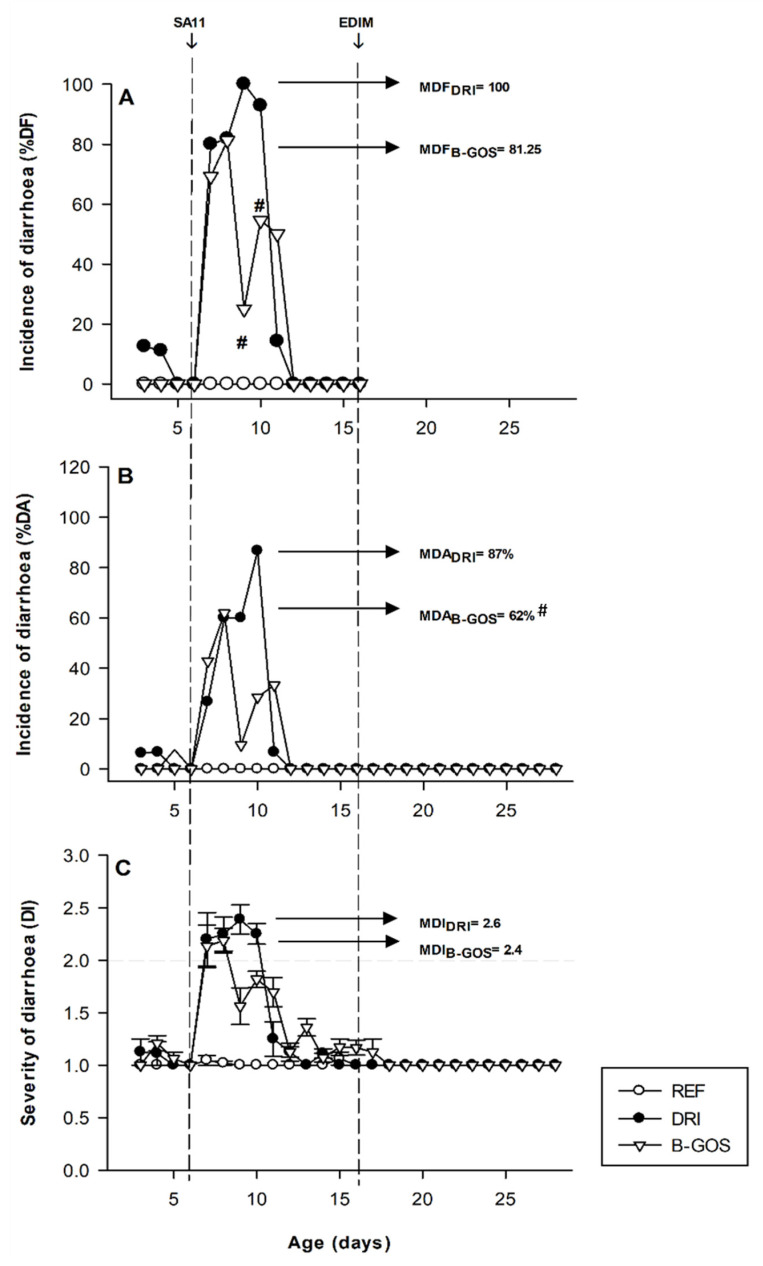
Incidence and severity of the diarrhea for reference (REF), double-rotavirus-infected (DRI), and prebiotic-supplemented (B-GOS) animals. Results are expressed as % of diarrheic feces (**A**), % of diarrheic animals (**B**), and the severity of diarrhea (**C**). Scores of diarrhea index (DI) ≥ 2 indicate diarrheic feces. The inoculation days are indicated in dashed lines. Results are expressed as the mean ± SEM of *n* = 12–21 values/group. Statistical differences: (**A**,**B**) ^#^ *p* < 0.05 vs. DRI, and (**C**) mentioned in the text.

The diarrhea only appeared after the first infection (day six with SA11) and no changes in fecal features were obtained after the second RV inoculation (day 17 with EDIM) (Figure 2). 

The incidence of SA11-induced diarrhea was expressed as the % DF (diarrheic feces from the total specimens obtained, Figure 2A) and the % DA (diarrheic feces obtained from all the animals, Figure 2B) and in both cases showed similar results. The DRI group contained animals displaying diarrhea from day 6 to day 11. In the B-GOS-supplemented group, a preventive effect was found during the diarrhea period. Thus, B-GOS significantly reduced the maximum proportion of animals with diarrhea, from 100 to 81.25% MDF and from 87 to 61.9% MDA. In both infected groups (DRI and B-GOS) the diarrhea disappeared at day 12.

The B-GOS group also has a reduction in the overall severity when compared to the DRI group (Figure 2C). B-GOS animals displayed 35 and 20% lower diarrhea scores than the DRI group on days 9 and 10 (*p* < 0.05), respectively. Although not significant, the AUC from the diarrhea score profile and the maximum DI from the B-GOS group was 20 and 10% lower than those from the DRI group, respectively. However, the mean number of days in which animals displayed diarrhea was reduced by B-GOS supplementation by 0.64 days (*p* < 0.05 vs. DRI).

### 3.3. Other Clinical Features

**Figure 3:**
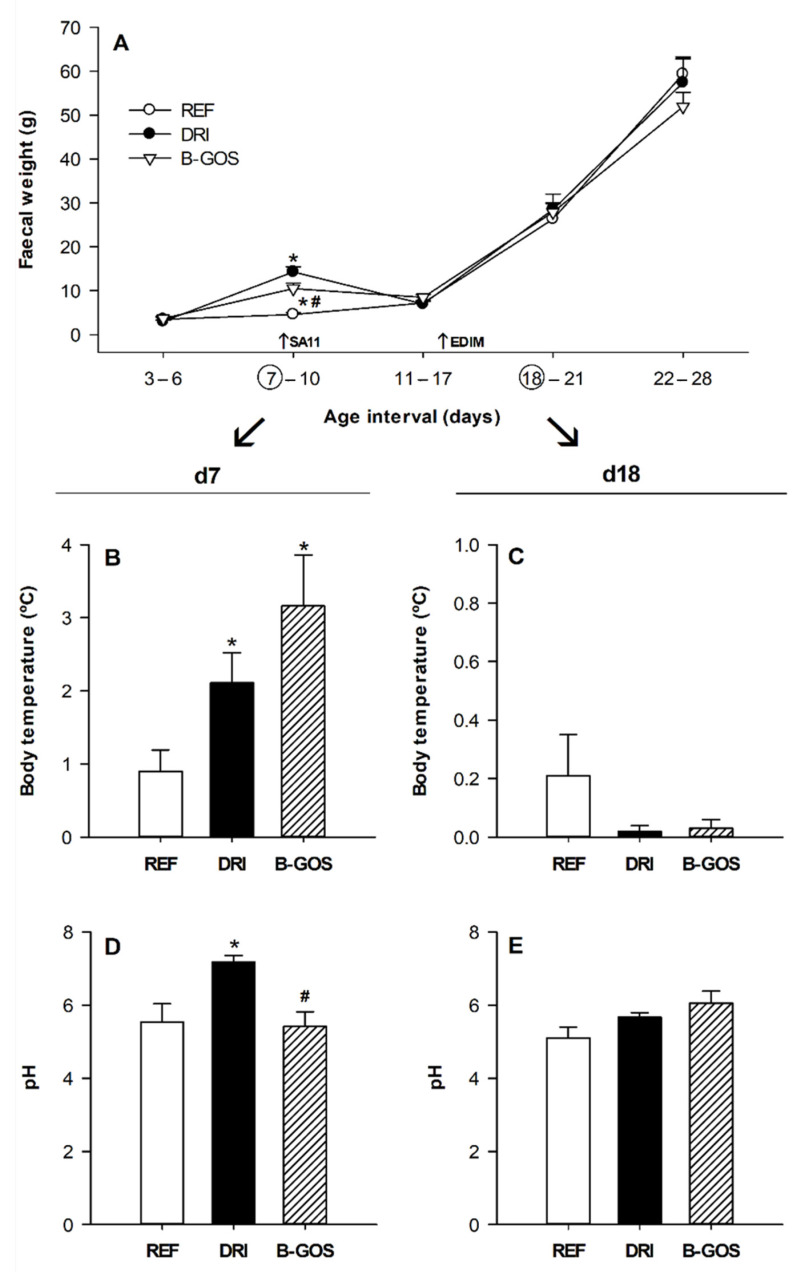
Clinical features derived from infection for reference (REF), double-rotavirus-infected (DRI), and prebiotic-supplemented (B-GOS) groups. Fecal weight during the studied period (**A**), rectal temperature on day 7 (**B**) and day 18 (**C**), and fecal pH on day 7 (**D**) and day 18 (**E**). Results are expressed as the mean ± SEM (*n* = 12–21 animals/group). Statistical differences: * *p* < 0.05 vs. REF, ^#^ *p* < 0.05 vs. DRI.

Complementary determinations, such as the fecal weight, the body temperature, and the fecal pH were also monitored after both SA-11 and EDIM inoculations (Figure 3).

The fecal sample weights recorded throughout the study showed that, in accordance with the diarrhea score, they only changed due to the first, but not the second, RV infection (Figure 3A). The mean fecal weight from the DRI animals during the acute diarrhea period (days 7–10) was 14.3 ± 1.2 mg whereas B-GOS supplementation significantly reduced it to 10.5 ± 1.2 mg (*p* < 0.05). Later, all groups displayed increasing fecal weight up to 50–60 mg on day 28, without any differences observed, which was due to either the infection or the supplementation.

After SA-11 infection, an increase in body temperature was observed in both the DRI and the B-GOS groups (*p* < 0.05) on day seven, the day after inoculation (Figure 3B). However, the EDIM inoculation did not have any effect on the rectal temperature the following day (Figure 3C).

The pH of the fecal samples was also determined the day after inoculation in both infections, at day 7 and 18, for SA-11 and EDIM, respectively. The SA-11 infection in the DRI group increased the fecal pH with respect to the REF group (*p* < 0.05), and this increase was prevented by the B-GOS supplementation (Figure 3D). The fecal pH on day 18 was not affected either by the EDIM inoculation or the B-GOS supplementation (Figure 3E).

### 3.4. Viral Shedding

The DRI and the B-GOS animals displayed the maximum viral shedding on the first day after inoculation for both SA-11 and EDIM infections, i.e., day 7 and day 18, respectively. With regard to the infection with SA-11, the B-GOS group showed a tendency to reduce 20 and 10% of the viral clearance on days seven and eight, respectively (*p* < 0.1). On the contrary, the viral shedding on days 18 and 19 had a tendency to increase about 30 and 15%, respectively, in the B-GOS with respect to the DRI group; again, this was without statistical significance due to the high interindividual variability found.

### 3.5. DTH Response

At the end of the study, the DTH response was analyzed. No changes were observed in the ear thickness of the injected ear after 24 and 48 h post-ear priming, the ear thickness increase with respect to the initial measure, or the difference between the injected and not-injected ear at each time between the DRI and B-GOS groups (data not shown).

### 3.6. Anti-RV Ab Response

**Figure 4:**
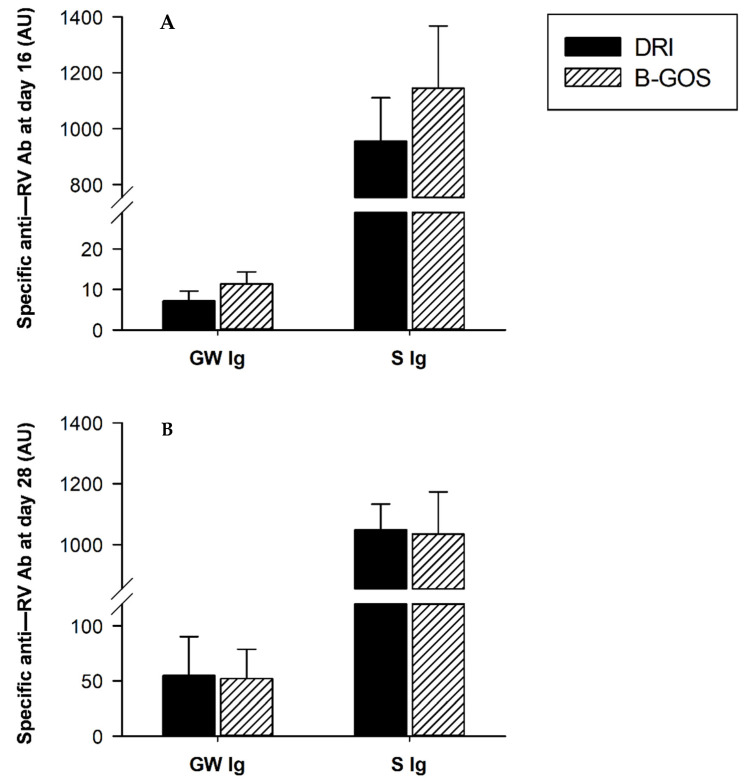
Specific anti-RV Ab in GW and serum from 16 (**A**) and 28 (**B**) day-old rats in double-rotavirus-infected (DRI) and prebiotic-supplemented (B-GOS) groups. Results are expressed as arbitrary units (AU) of specific Ab and represented as mean ± SEM (*n* = 6–12 animals/group).

Specific anti-RV Abs were quantified in 16- and 28-day-old rats (Figure 4). Anti-RV Ab at mucosal level (i.e., in the GW) and at systemic level (i.e., serum), were found at both time points studied: after the first infection (day 16, Figure 4A) and after the second infection (day 28, Figure 4B). The experimental intervention with B-GOS from day 3 to day 14 was not able to affect the anti-RV immune response in any of the compartments studied with respect to the DRI group, although a tendency to increase both the mucosal and the serum anti-RV Ab levels was found on day 16 of life, just prior to the second infection.

**Figure 5:**
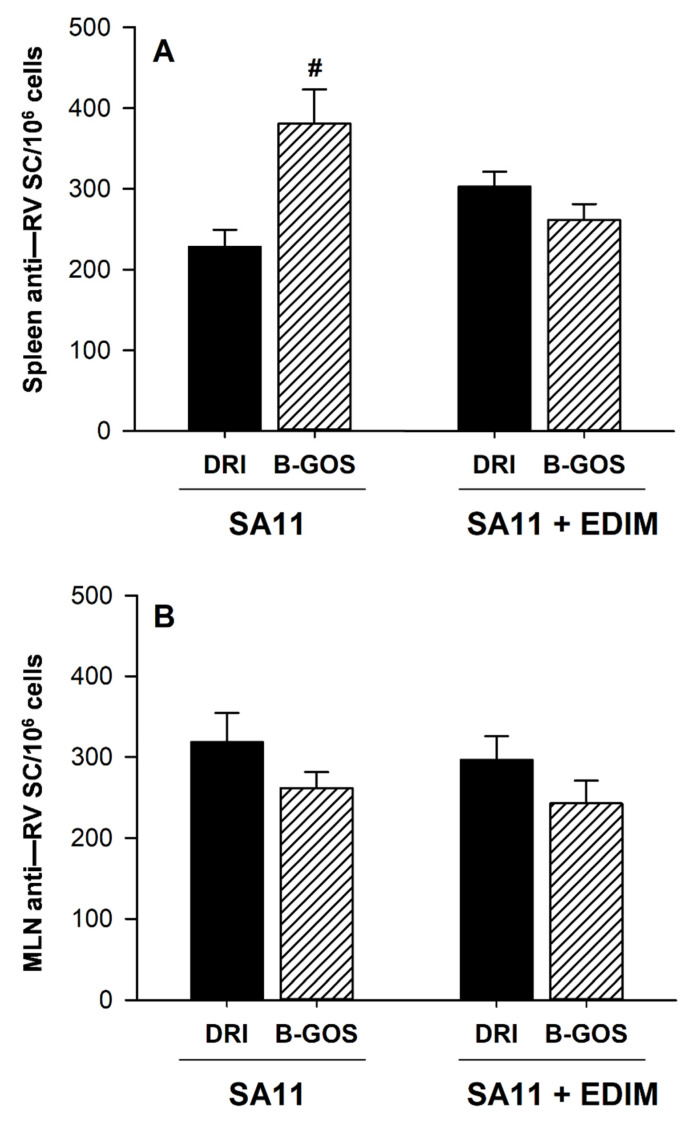
Ex vivo anti-RV Ig-secreting cells (SC) in the spleen (**A**) and MLN (**B**) from 28-day-old animals from double-infected groups without (DRI) or with dietary intervention (B-GOS). Results are expressed as the mean Ig-SC/10^6^ cells ± S.E.M (*n* = 12 animals/group). Statistical differences: ^#^ *p* < 0.05 vs. DRI.

In addition, the influence of the B-GOS supplementation on the ability of isolated cells to produce anti-RV Ab in the systemic (spleen) (Figure 5A) and mucosal (MLN) (Figure 5B) compartments was also studied by ELISPOT after both SA11 and EDIM infections. The number of anti-RV secreting cells (SC) in the spleen and the MLN in both groups was similar (∼300 anti-RV SC/10^6^ cells) with the exception of that from the spleen cells after the first infection (∼200 anti-RV SC/10^6^ cells), which was lower (*p* < 0.05, Figure 5). Apart from that, the B-GOS administration only induced a significant increase in the number of anti-RV Ig-SC in the spleen after the SA-11 infection (*p* < 0.05).

### 3.7. Gut Wash Levels and the Ex Vivo Production of Cytokines

As the RV infection may boost the cytokine production, their levels were analyzed in the GW and after 72 h culture of spleen ex vivo production from 28-day-old rats (Table 1).

In the GW, the infections in the DRI group induced an increase in the IFN-γ levels with respect to the REF group (*p* < 0.05). This increase was even higher in those animals supplemented with B-GOS (*p* < 0.05). IL-4, IL-10, and TNF-α levels were not influenced by the SA-11 and EDIM infections in the DRI group, but the B-GOS significantly induced higher levels than REF and/or DRI groups (*p* < 0.05). The enhancement in the production of IL-4 and TNF-α by B-GOS was also found in the spleen cells that were cultured during 72 h (*p* < 0.05).

### 3.8. Intestinal Gene Expression

**Figure 6:**
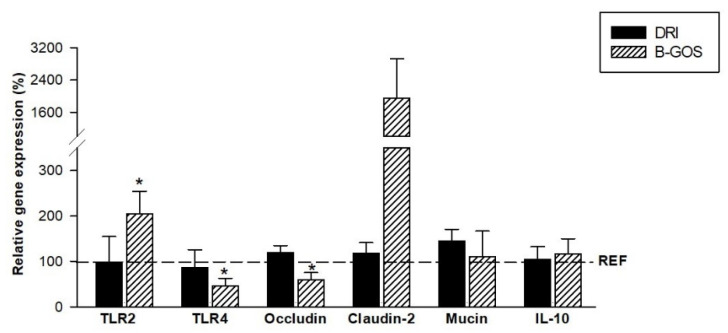
Intestinal gene expression of 28-day-old rats from double-rotavirus-infected (DRI) and prebiotic-supplemented (B-GOS) groups. Results are expressed as the mean relative gene expression of the reference group ± SEM (*n* = 6 animals/group). Statistical significance: * *p* < 0.05 vs. REF.

The intestinal gene expression of molecules involved in the immune response and intestinal barrier was evaluated at the end of the study (Figure 6). The animals from the DRI group, after the two infective processes (28-day-old), did not show modifications in any of the genes analyzed: in the TLRs, the tight junction genes, the mucin, or the cytokine IL-10. However, those animals receiving B-GOS just for 7 days during suckling affected some of their intestinal expression. Thus, the gene expression of TLR2 was significantly increased, whereas that of TLR4 and occludin were decreased (*p* < 0.05).

## 4. Discussion

The diarrhea associated with rotavirus infections in children can be acute and severe, and besides appropriate rehydration, the intake of prebiotics as preventive agents seems to be a good strategy. In this study, on the one hand, a prebiotic with anti-infective and immunomodulatory potential has been selected [23,24]. On the other hand, it has been tested in a double-RV infection model in neonatal rats, which reproduces the multiple contacts and/or infections produced in children, which is better than using the simple infection model [7]. In any case, the single and multiple rat infection models have been used satisfactorily in previous studies evaluating the effect of prebiotics, probiotics, symbiotics, and even postbiotics [7,27,29].

The first RV used in the current approach caused an acute diarrhea in the suckling rats that lasted for several days after the inoculation. In this model, it has been observed that the diarrhea is induced by the virus through the induction of watery loss in the feces, and therefore results in the increase in fecal weight, the induction of intestinal dysbiosis, and the alteration in the immune response and intestinal integrity of the host [7,16,29,33]. This mild diarrhea was not accompanied by BW loss, as already observed in previous studies [7,27,29], but by changes in the fecal weight and pH and an increase in rectal temperature due to the infection. However, in line with previous results [27], the second RV inoculation in older animals did not induce clinical symptoms [7], even when an early weaning close to the infection day was performed.

The administration of the prebiotic before the first infection and during the diarrhea period was enough to show its positive effects. The B-GOS supplementation lowered the incidence and also reduced the diarrhea period and the severity of the process. Although the diarrhea score on which these variables are based could be difficult to assess, its association with an objective variable such as the fecal weight validate this evidence. In fact, the prebiotic intervention was also able to prevent the fecal weight increase and even the change in fecal pH caused by the SA-11 inoculation. However, it could not revert the rise in temperature. This preventive effect on the SA-11-associated diarrhea was similar to that obtained with a mixture of long-chain fructo-oligosaccharides (lcFOS) and short-chain galacto-oligosaccharides (scGOS) [16,27].

One of the mechanisms suggested for the direct action of prebiotics is their binding to the pathogen, which allows its better elimination. This mechanism that has been demonstrated for other prebiotics in this model [27,33] does not seem to be involved in the present study, because the viral shedding was not affected after the B-GOS intervention. Moreover, the modulation of the diarrhea did not avoid the generation of the appropriate anti-viral humoral immune response, as the levels of anti-RV Ab were similar to those in the non-supplemented group. Overall, it can be suggested that the B-GOS ameliorated the diarrhea process at a clinical level without affecting the generation of primed immune cells and, therefore, the antibody response, which could be required for further infections.

As stated, the second infection did not display any quantifiable symptoms, according to previous results [7,27]; thus, the effect of the prebiotic intervention could not be evaluated during the early post-infection period by the analysis of fecal weight or pH, the viral shedding, or the rectal temperature. However, it must be considered that the present study was aimed to explore if the modulation of the first infection with the B-GOS permits an appropriate immune response against a second RV infection later in life.

The generation of the mucosal and systemic humoral immune response, in terms of anti-RV Ab, was not affected by the prebiotic intervention during the first infection. This is in line with a previous study using a scGOS/lcFOS mixture [27], which also modulated the diarrhea from the first infection but did not influence the anti-RV Ab levels after a second infection. However, the capacity of spleen cells from 28-day-old animals, with a more developed immune system than the suckling animals, was enhanced by the B-GOS supplementation earlier in life, from day 3 to day 14. This fact suggests the immunomodulatory role of this prebiotic that seems to reinforce the ability to produce Ab, at least at a systemic level, as no changes were observed in the MLN. In fact, this lack of effect at the mucosal level agrees with the absence of changes in secretory IgA due to B-GOS supplementation that are observed in elderly people [23].

In the model’s set-up, it was shown that the ex vivo cytokine production was different between single- or double-infected animals, evidencing that the first infection induced different immune responses than in the second infection [7,27]. Moreover, the intervention with the lcFOS/scGOS mixture in this model pointed out a potentiation in the production of some cytokines [27]. In the present study, the B-GOS also induced changes in the cytokine levels in the spleen and MLN ex vivo assays and more notoriously in the GW. Particularly, the B-GOS supplementation enhanced the T helper 2 (Th2) immunity by inducing higher levels of IL-4, which is aligned to the Ab synthesis. Furthermore, the B-GOS also increased the TNFα production in both approaches. In addition, at the intestinal compartment, the anti-inflammatory cytokine IL-10 and the antiviral cytokine IFNγ were also increased. The potentiating effect on IL-10 observed here is in line with that observed in peripheral blood mononuclear cells (PBMC) from elderly subjects supplemented with B-GOS for 10 weeks [23].

Although it was not studied in the present study, it could also be suggested that the immunomodulatory role of B-GOS, and therefore the immune response against the virus, could be mediated through the action of the NK cells, as this prebiotic has shown its enhancing effect on NK cell activity in PBMC [23].

Moreover, the intestinal gene expression after the two RV inoculations was not modified by the infective processes, as demonstrated in the previous studies [27]. However, some changes were observed to be due to the prebiotic intervention. Specifically, although other studies described the ability of some bacteria and bacterial products to improve the barrier function [34], in the present work the expression of occludin was downmodulated, whereas that of claudin tended to be enhanced. Although the interpretation of the tight junction proteins’ modulation is not clear, this effect was also found in this same model with another prebiotic intervention, thus, confirming this awkward effect, which deserves to be further studied.

The microbiota-derived metabolites can activate TLR2 on glial cells, which results in an increased IL-22 secretion by innate lymphoid cells (ILC)-3 to provide protection against inflammation or pathogenic infection [4]. Thus, the significant increase by B-GOS of the intestinal gene expression of TLR2 could be linked to the possible effects of the prebiotic on the microbiota composition, which has not been analyzed in the present study. In line with this, the RV infection can cause dysbiosis and alter the TLR gene expression [35], however these changes were observed only during the peak of the RV-induced diarrhea and without knowing how long this effect lasted. In addition, it would have been interesting to study the changes in the microbiota composition due to each RV infection and the role of B-GOS in each situation. It is plausible to think that some of the beneficial effects ascribed here to the B-GOS on its diarrhea prevention and on its immune system modulation could be microbiota-dependent, and this should be addressed in the future. In fact, microbiota-dependent and -independent mechanisms have been described in animals and in humans after prebiotic interventions to treat intestinal infections [11]. However, further studies should be performed to better stablish the appropriate posology in each case and to obtain more solid evidence of their preventive potential. An existing limitation of this type of research is that a combination of prebiotics and probiotics is often used, which does not allow to elucidate the effect of each compound alone.

Finally, it is known that the ENS is activated and plays a key role in the RV pathogenesis, with serotonin and its receptors being one of the main involved signals [36]. In this regard, the activation of the ENS by the RV stimulates the enterochromaffin cells to release serotonin (5-HT) and, therefore, it activates the secretomotor pathway, leading to propulsive intestinal motility [4]. Moreover, it is known that some microbiota members can produce diverse microbial metabolites that can influence host serotonergic systems in a variety of ways [37], thus it can be plausible to think that this mechanism could also be involved in the positive effects of the nutritional intervention performed, and this should be approached in detail in future experimental designs.

## 5. Conclusions

Overall, B-GOS supplementation for just some days in early life was enough to reduce the incidence and severity of the RV-associated diarrhea. This effect does not seem to affect the development of the appropriate immune response against the virus later in life, however, it showed some beneficial immunomodulatory activity. As only a few mediators were evaluated, more studies are required to prove the effect on the immune system and the mechanisms involved. Thus, this prebiotic seems to be an attractive component to be added to infant formulas and to help non-breastfed babies to counteract this prevalent infection.

## Figures and Tables

**Table 1:** Cytokine levels in GW and after 72 h ex vivo production by spleen cells obtained from 28-day-old rats from reference (REF), double-rotavirus-infected (DRI), and prebiotic-supplemented (B-GOS) groups. Results are expressed as mean ± SEM (*n* = 3–12 animals/group). Statistical significance: * *p* < 0.05 vs. REF, ^#^ *p* < 0.05 vs. DRI.

	Gut Wash			72 h Spleen Cell Supernatants		
	REF	DRI	B-GOS	REF	DRI	B-GOS
IL-4 (pg/mL)	6.18 ± 0.60	6.15 ± 1.05	16.03 ± 0.42 *^#^	0.42 ± 0.31	0.33 ± 0.26	3.34 ± 0.52 *^#^
IL-10 (ng/mL)	38.02 ± 23.42	160.00 ± 70.13	311.98 ± 58.96 *	1.27 ± 3.5	1.18 ± 0.23	1.82 ± 0.27
IFN-γ (pg/mL)	n.d.	20.63 ± 7.26 *	69.73 ± 12.59 *^#^	n.d	n.d.	1.72 ± 0.89
TNF-α (pg/mL)	n.d.	n.d.	301.95 ± 43.63 *^#^	91.43 ± 48.65	30.12 ± 16.18	324.21 ± 45.68 *^#^
